# PRMT5 Enables Robust STAT3 Activation via Arginine Symmetric Dimethylation of SMAD7

**DOI:** 10.1002/advs.202003047

**Published:** 2021-02-24

**Authors:** Congcong Cai, Shuchen Gu, Yi Yu, Yezhang Zhu, HanChenxi Zhang, Bo Yuan, Li Shen, Bing Yang, Xin‐Hua Feng

**Affiliations:** ^1^ The MOE Key Laboratory of Biosystems Homeostasis & Protection and Innovation Center for Cell Signaling Network, Life Sciences Institute Zhejiang University Hangzhou Zhejiang 310058 China; ^2^ The Key Laboratory of Cancer Molecular Cell Biology of Zhejiang Province, Life Sciences Institute Zhejiang University Hangzhou Zhejiang 310058 China; ^3^ The Second Affiliated Hospital Zhejiang University Hangzhou Zhejiang 310009 China

**Keywords:** methylation, migration, PRMT5, proliferation, Smad7, STAT3

## Abstract

Protein arginine methyltransferase 5 (PRMT5) is the type II arginine methyltransferase that catalyzes the mono‐ and symmetrical dimethylation of protein substrates at the arginine residues. Emerging evidence reveals that PRMT5 is involved in the regulation of tumor cell proliferation and cancer development. However, the exact role of PRMT5 in human lung cancer cell proliferation and the underlying molecular mechanism remain largely elusive. Here, it is shown that PRMT5 promotes lung cancer cell proliferation through the Smad7‐STAT3 axis. Depletion or inhibition of PRMT5 dramatically dampens STAT3 activation and thus suppresses the proliferation of human lung cancer cells. Furthermore, depletion of Smad7 blocks PRMT5‐mediated STAT3 activation. Mechanistically, PRMT5 binds to and methylates Smad7 on Arg‐57, enhances Smad7 binding to IL‐6 co‐receptor gp130, and consequently ensures robust STAT3 activation. The findings position PRMT5 as a critical regulator of STAT3 activation, and suggest it as a potential therapeutic target for the treatment of human lung cancer.

## Graphical abstract

The role of protein arginine methyltransferase 5 (PRMT5) in Janus kinase (JAK)‐signal transducer and activator of transcription 3 (STAT3) signaling pathway and lung cancer cell proliferation is unveiled. PRMT5 symmetrically dimethylates Smad7 on Arginine‐57, promotes Smad7 binding to interleukin‐6 (IL‐6) co‐receptor glycoprotein 130 (gp130), and consequently ensures robust STAT3 activation in a transforming growth factor‐*β* (TGF‐*β*) independent manner. This finding positions PRMT5 as a promising therapeutic target for lung cancer treatment.

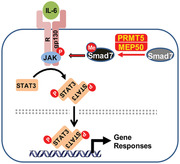

## 1 Introduction

Protein arginine methyltransferase 5 (PRMT5) is a type II arginine methyltransferase that catalyzes the mono‐ and symmetrical dimethylation of protein substrates at the arginine residues.^[^ ^1^ ^]^ It was first identified as a JAK2 binding protein.^[^ ^2^ ^]^ PRMT5 methylates arginine residues in histones and epigenetically controls the expression of an array of target genes.^[^ ^3^, ^4^ ^]^ In addition, PRMT5 also modifies non‐histone proteins to control their functions, including transcription factors p53,^[^ ^5^ ^]^ KLF4,^[^ ^6^ ^]^ E2F‐1,^[^ ^7^, ^8^ ^]^ NF‐*κ*B/p65,^[^ ^8^ ^]^ ribosomal protein S10,^[^ ^9^ ^]^ and RAF kinases.^[^ ^10^ ^]^ PRMT5 is ubiquitously expressed in all eukaryotes and is involved in a wide range of biological processes, such as germ cell survival,^[^ ^11^ ^]^ cell cycle progression,^[^ ^12^ ^]^ muscle stem cell expansion,^[^ ^13^ ^]^ and myogenesis.^[^ ^14^ ^]^ In addition to these functions, PRMT5 plays a pivotal role in cancer cell proliferation and transformation.^[^ ^15^ ^]^ Increased expression of PRMT5 has been observed in a variety of carcinomas, including ovarian, lung, lymphoma, melanoma, colon, gastric, bladder cancer, and germ cell tumors.^[^ ^16^ ^]^. Methylosome protein 50 (MEP50, also known as WDR77) is a critical cofactor of PRMT5, both with similar expression pattern.^[^ ^17^ ^]^ MEP50 binds to PRMT5 and greatly enhances its catalytic ability.^[^ ^18^, ^19^ ^]^ PRMT5 also directly associates with a range of other protein factors, including pICln, Menin, CoPR5, and RioK1, which may alter its subcellular localization and protein substrate selection.^[^ ^20^, ^21^, ^22^ ^]^

The IL‐6/JAK/STAT3 pathway has a key role in the growth and development of many human cancers.^[^ ^23^, ^24^ ^]^ Elevated levels of IL‐6 are observed in a large number of patients with hematopoietic malignancies or solid tumors.^[^ ^23^, ^25^ ^]^ Aberrantly increased IL‐6 stimulates hyperactivation of JAK/STAT3 signaling, which is often associated with poor patient outcomes. In the tumor microenvironment, IL‐6/JAK/STAT3 signaling acts to drive the proliferation, survival, invasiveness, and metastasis of tumor cells. ^[^ ^26^ ^]^ In the classic IL‐6/JAK/STAT3 signaling, IL‐6 binds to the membrane‐bound IL‐6 receptor‐*α* (IL‐6R*α*), thus inducing the formation of a heterohexameric complex with gp130 (also known as IL‐6R*β*). Formation of this complex results in JAK2 activation and STAT3 phosphorylation. Then phosphorylated STAT3 becomes dimerized, transports into the nucleus and binds to STAT‐binding elements in the promoter regions of target genes.^[^ ^24^ ^]^ Although PRMT5 is first identified as a JAK2 interacting protein, until now PRMT5 substrates have not been identified in the JAK complex.^[^ ^27^, ^28^ ^]^ Moreover, it still remains unknown whether or how PRMT5 affects JAK/STAT signaling.

In this study, we identified and characterized PRMT5 as an essential mediator of STAT3 activation. Depletion or inhibition of PRMT5 dramatically dampens STAT3 activation and suppresses proliferation and migration of human lung cancer cells. Moreover, we uncovered that PRMT5 promotes STAT3 activation through methylation of Smad7 on Arg‐57. We previously reported that Smad7 directly binds to the intracellular domain of gp130, thereafter disrupting the SHP2‐ or SOCS3‐gp130 complex and amplifying STAT3 activation. We now provide evidence that methylated Smad7 exhibits a stronger interaction with gp130. Our findings identified the oncogenic role of PRMT5 in human lung cancer and elucidated a novel mechanism underlying the modulation of the Smad7‐gp130‐STAT3 axis by PRMT5 in those cancer types with aberrantly hyperactivation of STAT3.

## 2 Results

### 2.1 PRMT5 Promotes STAT3 Activation through Smad7

**Figure 1:**
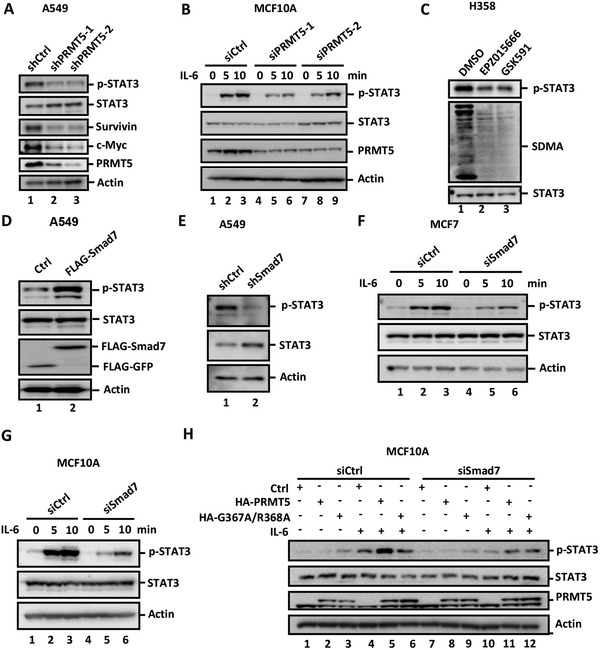
PRMT5 potentiates STAT3 activation via Smad7. A) PRMT5 depletion dampens endogenous STAT3 activation in A549 cells. A549 cells stably expressing shPRMT5‐1 or shPRMT5‐2 or Control (shCtrl) were harvested and analyzed by using western blotting with indicated antibodies. B) Knockdown of PRMT5 attenuates IL‐6‐induced STAT3 phosphorylation in MCF10A cells. MCF10A cells were transfected with 40 pm siRNA against PRMT5. 36 h later, cells were treated with IL‐6 (10 ng mL^−1^) for the indicated time and harvested for western blotting analysis with appropriate antibodies. C) PRMT5 inhibition attenuates endogenous activation of STAT3 in H358 cells. H358 cells were treated with 20 × 10^−6^ m of PRMT5 inhibitors EPZ015666 or GSK591 for the indicated time. Cell lysates were collected and subject to western blotting analysis. SDMA indicates global arginine di‐methylation. D) Smad7 potentiates STAT3 activation in A549 cells. A549 cells stably expressing FLAG‐GFP or FLAG‐Smad7 were harvested and subject to Western blotting analysis using appropriate antibodies. E) Stable knockdown of Smad7 dampens endogenous STAT3 activation in A549 cells. A549 cells stably expressing shSmad7 or shCtrl were harvested and subject to western blotting analysis using appropriate antibodies. F) Smad7 depletion dampens IL‐6‐induced STAT3 activation in MCF7 cells. Cells were transfected with siSmad7 (40 pm) and treated with IL‐6 (10 ng mL^−1^) for the indicated time. Cells were harvested and analyzed by western blotting with appropriate antibodies. G) Smad7 depletion dampens IL‐6‐induced STAT3 activation in MCF10A cells. Cell transfection, treatment, and Western blotting were done as described in Panel F. H) PRMT5 potentiates STAT3 activation dependent of Smad7. MCF10A cells were transduced with lentiviral particles expressing HA‐PRMT5 or HA‐G367A/R368A. After 24 h, cells were transfected with 40 pm siSmad7. 12 h later, cells were stimulated with IL‐6 (2 ng mL^−1^) for the indicated time. Cell lysates were harvested and subject to Western blotting analysis using appropriate antibodies.

PRMT5 was previously identified as a JAK2‐interacting protein,^[^ ^2^ ^]^ implicating that PRMT5 may regulate STAT3 signaling. However, it remains unknown whether PRMT5 acts through STAT3 signaling in growth regulation and tumorigenesis. In this study, we sought to address this long‐standing question on the potential connection between PRMT5 and STAT3 signaling. To this end, we first utilized siRNAs/shRNAs against two different target sequences to knockdown the expression of PRMT5 and then examined the status of STAT3 activation, as indicated by Tyr‐705 phosphorylation. Stable depletion of PRMT5 profoundly reduced endogenous STAT3 phosphorylation and expression of two STAT3 targets, e.g., survivin and c‐Myc, in lung carcinoma A549 cells (**Figure** **1**A). Similar results were observed in another lung carcinoma cell line H358 (Figure S1A, Supporting Information), suggesting that PRMT5 might potentiate STAT3 activation induced by autocrine IL‐6 in lung carcinoma cells. Both A549 and H358 cells secreted a high level of IL‐6 (Figure S1B, Supporting Information), and anti‐IL‐6 receptor (IL‐6R) antibody tocilizumab blocked STAT3 activation in A549 cells (Figure S1C, Supporting Information). When the A549‐conditioned medium was applied to human mammary epithelial cells MCF10A, STAT3 was potently activated, which could also be blocked by tocilizumab (Figure S1D, Supporting Information). To further test the role of PRMT5 in exogenous IL‐6‐induced STAT3 activation, PRMT5 was depleted in MCF10A cells. As shown in Figure 1B, MCF10A cells were responsive to IL‐6, and exhibited induced STAT3 Y705 phosphorylation upon ligand stimulation, whereas transient knockdown of PRMT5 reduced STAT3 phosphorylation stimulated by IL‐6. Furthermore, the RNAi‐resistant variant of PRMT5 (PRMT5r) could rescue siPRMT5‐mediated suppression on IL‐6‐stimulated STAT3 activation in MCF10A cells (Figure S1E, Supporting Information compare lane 8–9 to lane 5–6). These data suggest that PRMT5 promotes robust STAT3 activation in response to IL‐6.

PRMT5 is a protein methyltransferase that is responsible for the vast majority of cellular symmetric dimethylation of arginine (SDMA) modification. We then examined whether the methyltransferase activity of PRMT5 is essential for STAT3 activation by using chemical inhibitors of PRMT5. Treatment of H358 and A549 cells with EPZ015666 or GSK591, two recently developed potent and specific PRMT5 chemical inhibitors,^[^ ^30^, ^31^ ^]^ lead to a significant loss of PRMT5‐catalyzed symmetrically dimethylated proteins, as revealed by Western blotting using a pan‐SDMA antibody (sdme‐RG) that recognized a subset of cellular proteins with symmetrical dimethylation at arginine residues (Figure 1C and Figure S1F, Supporting Information). Notably, these inhibitors caused the reduction of endogenous STAT3 phosphorylation in H358 cells (Figure 1C) and A549 cells (Figure S1F, Supporting Information) as well as IL‐6‐stimulated MCF10A cells (Figure S1G, Supporting Information). In the RNAi rescue experiment, unlike wildtype PRMT5, a catalytically inactive mutant of PRMT5, with glycine and arginine to alanine substitution at amino acid residues 367 and 368 (G367A/R368A),^[^ ^4^ ^]^ failed to exhibit the rescuing effect (Figure S1E, Supporting Information, lane 11–12). These results suggest that the methyltransferase activity strongly support a direct role of PRMT5 in STAT3 activation.

Since we recently reported that Smad7 promotes LIF‐induced STAT3 activation in mouse embryonic stem cells (mESC),^[^ ^32^ ^]^ we reasoned that Smad7 might be involved in PRMT5‐regulated STAT3 activation in epithelial cells. We first determined if Smad7 could promote STAT3 signaling in non‐mESC cells and/or in response to a non‐LIF cytokine. Like in mESC, overexpression of Smad7 could enhance STAT3 activation in lung carcinoma A549 cells (Figure 1D). Conversely, depletion of Smad7 nearly abolished STAT3 activation in A549 cells (Figure 1E and Figure S2A,B, Supporting Information). Thus, similarly to PRMT5, Smad7 promoted STAT3 activation perhaps through enhancement of autocrine IL‐6 signaling in A549 cells. We then tested the effect of Smad7 on STAT3 activation in response to exogenous IL‐6. Expression of Smad7 could enhance IL‐6‐induced STAT3 activation in MCF7 cells and MCF10A cells, both of which stably express Smad7 (Figure S2C,,D, Supporting Information). In contrast, an obvious reduction of IL‐6‐induced STAT3 phosphorylation was observed in MCF7 (Figure 1F) and MCF10A cells (Figure 1G), when Smad7 was depleted (Figure S2E,F, Supporting Information).

To connect Smad7 to PRMT5 on STAT3 activation, we determined whether Smad7 mediates PRMT5‐mediated STAT3 activation. In control cells, ectopic expression of PRMT5, but not the methyltransferase‐dead mutant G367A/R368A, enhanced IL‐6‐induced STAT3 activation (Figure 1H). Notably, knockdown of Smad7 markedly attenuated the stimulatory effect of PRMT5 on IL‐6‐induced STAT3 activation (Figure 1H). Moreover, knockdown of PRMT5 in T*β*RI‐null HaCaT cells could still inhibit the STAT3 activation (Figure S2G,H, Supporting Information). Taken together, these results suggest that PRMT5 stimulates JAK/STAT3 signaling dependent on Smad7, but independent of TGF‐*β* signaling.

### 2.2 PRMT5 Interacts with Smad7

**Figure 2:**
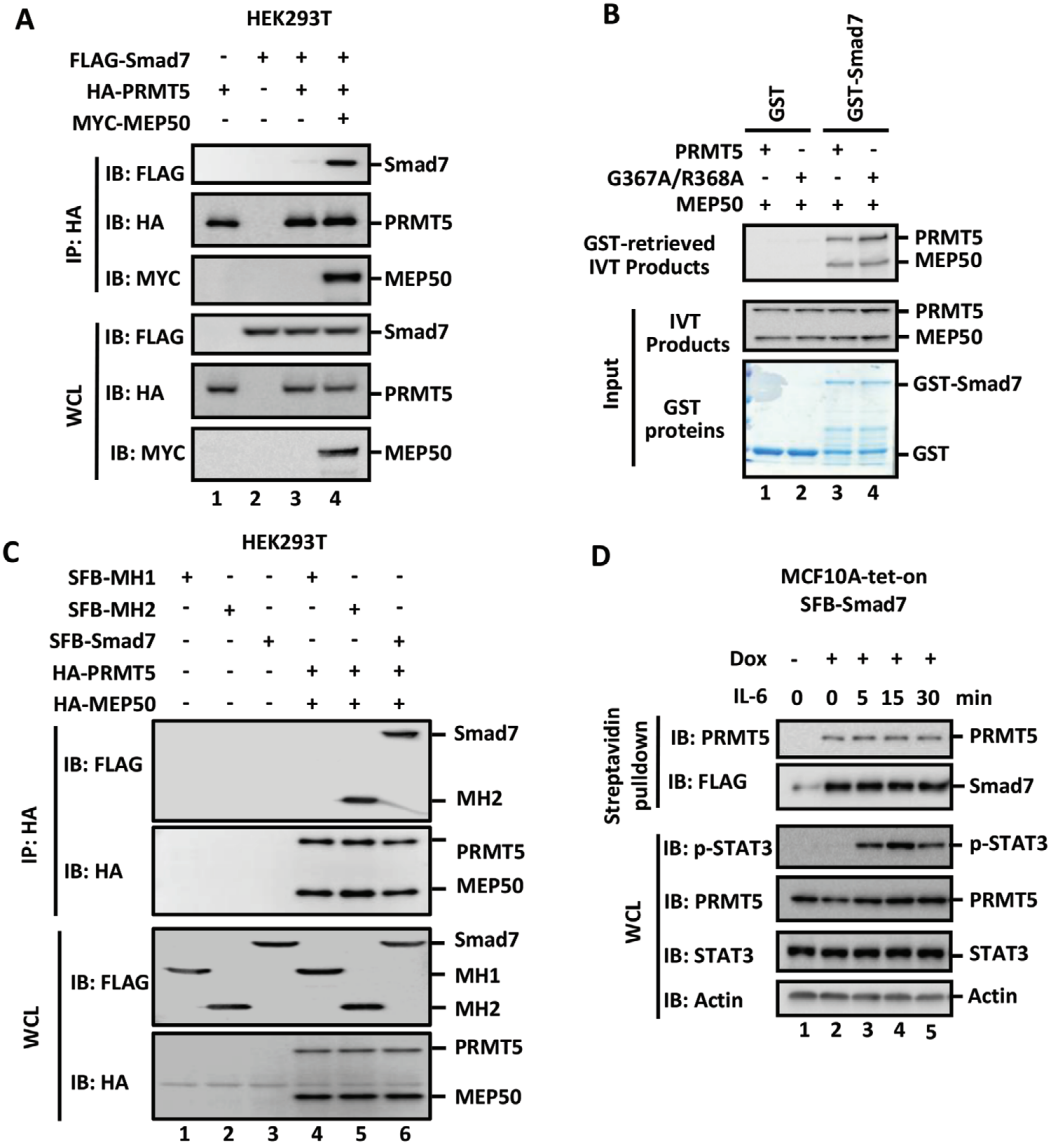
PRMT5 and MEP50 interact with Smad7. A) Smad7 interacts with PRMT5 and requires MEP50. HEK293T cells were transfected with HA‐PRMT5, FLAG‐Smad7, and MYC‐MEP50. Cell lysates were harvested and immunoprecipitated with HA antibody. The immunocomplexes and input were analyzed by using Western blotting analysis with indicated antibodies. B) Smad7 interacts with PRMT5 and MEP50 in vitro. Recombinant GST‐Smad7 or GST protein was produced and purified from *Escherichia coli*. MYC‐PRMT5 or G367A/R368A mutant together with MEP50 were expressed in HEK293T cells. In the GST pulldown assay, MYC‐PRMT5 or G367A/R368A proteins bound to GST proteins were retrieved with glutathione sepharose beads, and then analyzed by using Western blotting. C) PRMT5/MEP50 interacts with Smad7 in the MH2 domain. HEK293T cell transfection and Western blotting analysis were similarly done as described in Panel A. D) Smad7 interacts with endogenous PRMT5. Expression of SFB‐Smad7 was induced with or without 500 ng mL^−1^ Dox for 3 d in MCF10A‐tet‐on cells, and treated with 25 ng mL^−1^ IL‐6 for the indicated time. Cell lysates were harvested, precipitated with streptavidin beads, and analyzed by using Western blotting.

Having established that PRMT5 activated STAT3 signaling through Smad7, we assessed the potential interaction between PRMT5 and Smad7 by using coimmunoprecipitation (co‐IP) in HEK293T cells. We found that although PRMT5 is barely bound to Smad7, the PRMT5 cofactor MEP50 enabled a significantly higher PRMT5‐Smad7 interaction (**Figure** **2**A). To evaluate whether such interaction is direct, we conducted an in vitro binding assay. As shown in Figure 2B, recombinant GST‐Smad7 fusion protein, but not GST alone, could bind to immunopurified PRMT5 and MEP50, indicating that PRMT5 directly binds to Smad7. We also determined the domain of Smad7 that interacted with PRMT5. Wildtype Smad7 and Smad7‐MH2 (aa 228‐426) could interact with PRMT5, whereas the MH1 domain (aa 1‐228) alone failed to associate with PRMT5 (Figure 2C, Figure S2I, Supporting Information). Our results suggest that the MH2 domain of Smad7 is necessary for Smad7 binding to PRMT5.

To further investigate the PRMT5–Smad7 interaction under physiological conditions, we examined the binding of endogenous PRMT5 to Smad7. In the absence of a good anti‐Smad7 antibody for IP, we established stable tet‐on cell lines to inducibly express SFB‐Smad7 in MCF10A cells. In the Smad7‐tet‐on cells, doxycycline (Dox) treatment induced expression of SFB‐Smad7 (Figure S2J, Supporting Information). Dox‐induced Smad7 could interact with endogenous PRMT5, whereas no Smad7‐bound PRMT5 was detected in the absence of Dox (Figure 2D). Furthermore, IL‐6 stimulation had no effect on the interaction (Figure 2D).

### 2.3 PRMT5 Methylates Smad7 on Arg57

**Figure 3:**
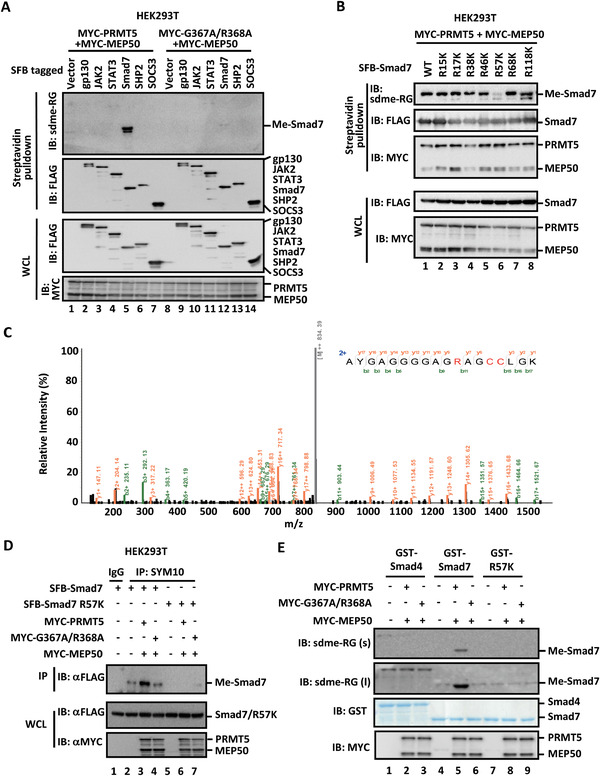
PRMT5 methylates Smad7 on R57. A) PRMT5 methylates Smad7. HEK293T cells were transfected with expression plasmids carrying MYC‐PRMT5/MEP50 and an SFB‐tagged construct, including gp130, JAK2, STAT3, Smad7, SHP2, and SOCS3. Cell lysates were harvested and precipitated with streptavidin beads. The retrieved complexes and input were analyzed by Western blotting with indicated antibodies. B) PRMT5 methylates the R57 residue on Smad7. HEK293T cells were transfected with MYC‐PRMT5/MEP50 and an SFB‐tagged Smad7 construct, i.e., wildtype Smad7 (WT) or a R‐to‐K substitution of Smad7 as indicated above the blots. Cell lysate was precipitated with streptavidin beads. Arginine di‐methylation of Smad7 was detected by Western blotting analysis. C) Mass spectrum of Smad7 Arg‐57 dimethylated peptide. Mass spectrometry identified Arg‐57 dimethylation of Smad7 in HEK293T cells expressing MYC‐PRMT5/MEP50 and SFB‐Smad7. Mass spectrometry profile of Smad7 sequence covering residue 47–64 is shown, and the dimethylated arginine side chains are indicated. D) PRMT5/MEP50 methylate Smad7, but not the R57K mutant. HEK293T cells were transfected MYC‐PRMT5/MEP50 and SFB‐Smad7 or Smad7 R57K mutant for 36 h. Cell lysates were harvested and immunoprecipitated with SYM10 antibody. The immunocomplexes and inputs were analyzed by Western blotting with indicated antibodies. E) PRMT5 methylates Smad7 in vitro. MYC‐PRMT5 or MYC‐G367A/R368A together with MYC‐MEP50 were immunopurified using anti‐MYC antibody from transfected HEK293T cells. Purified recombinant GST‐Smad7, GST‐Smad7 R57K mutant, and GST‐Smad4 were produced in *E. coli*. GST proteins and MYC‐PRMT5/MEP50 proteins were incubated in the presence of S‐adenosyl‐methionine to allow methylation reaction. Dimethylated Smad7 on R57 was detected by using Western blotting analysis.

Despite being identified as a JAK2‐interacting protein 20 years ago, PRMT5 has not been reported to methylate JAK kinases or STAT3.^[^ ^27^, ^28^ ^]^ We next attempted to determine whether PRMT5 could methylate any other components in the canonical JAK/STAT3 signaling pathway. We examined PRMT5‐mediated methylation in cells cotransfected with MYC‐PRMT5/MEP50 together with key components of the STAT3 signaling pathway, including SFB‐tagged JAK2, gp130, STAT3, SHP2, and SOCS3 as well as Smad7. Interestingly, PRMT5 could only methylate Smad7, but not others, as detected by sdme‐RG antibody (**Figure** **3**A, lane 5), whereas methyltransferase‐dead mutant G367A/R368A failed to methylate Smad7 (Figure 3A, lane 12). Moreover, Smad7 could only be methylated by PRMT5 in the presence of MEP50 (Figure S3A, Supporting Information), consistent with the interaction result (Figure 2A).

We next mapped which domain of Smad7 is methylated by PRMT5. In cells transfected with PRMT5/MEP50 and a Smad7 variant, we found that PRMT5 methylated Smad7 in the MH1 region that spans amino acids 1‐228 (Figure S3B, Supporting Information). To further delineate this, we constructed two molecular chimeras between Smad6 and Smad7, which contained the MH1 (or N) domain of one Smad and the MH2 (or C) domain of the other as previously described^[^ ^33^, ^34^ ^]^ (Figure S3C, Supporting Information). Smad6 is another inhibitory Smad in the TGF‐*β* superfamily with a C terminus similar to that of Smad7.^[^ ^35^ ^]^ We found that only Smad7 and Smad7/6 chimera, but not Smad6 or Smad6/7 chimera can be methylated by PRMT5 (Figure S3D, Supporting Information). These data point out that PRMT5 methylates the MH1 domain of Smad7.

To precisely localize the methylation site on Smad7, site mutagenesis was carried out on arginine residues that are adjacent to glycine (i.e., Gly‐Arg or GR) in the MH1 domain of Smad7. A GR motif represents putative preferred methylation sites of PRMT5.^[^ ^36^ ^]^ Each arginine within the MH1 domain was individually replaced by lysine, and coexpressed with PRMT5/MEP50 in HEK293T cells. Methylation of Smad7 variants was evaluated by streptavidin precipitation and Western blotting. Out of seven point mutations, only the R57K mutation abolish Smad7 methylation by PRMT5/MEP50 (Figure 3B). In addition, we performed mass spectrometry analysis of immunopurified Smad7 in HEK293T cells expressing MYC‐PRMT5/MEP50. A series of high mass accuracy y ions and b ions identified symmetric dimethylation on arginine‐57 (Arg‐57 or R57) of Smad7 (Figure 3C). Immunoprecipitation using SYM10 antibody (against sdme‐R) could effectively pull down methylated Smad7 in the presence of PRMT5/MEP50, but not G367A/R368A, whereas the R57K mutant could not be methylated by PRMT5/MEP50 (Figure 3D).

In an in vitro methylation assay, immuno‐purified MYC‐PRMT5/MEP50 proteins were incubated with bacterially expressed Smad7 (Figure 3E) or immunopurified SFB‐Smad7 proteins (Figure S3E,F, Supporting Information). It was apparent that PRMT5, but not G367A/R368A, methylated recombinant Smad7 (Figure 3E and Figure S3F, Supporting Information). On the contrary, PRMT5 could not methylate recombinant Smad7 R57K mutant or recombinant Smad4 (Figure 3E), further demonstrating that PRMT5 mediates specific methylation on R57 of Smad7. All of these analyses support the notion that PRMT5 specifically methylates Smad7 on Arg‐57 residue.

### 2.4 Arginine Methylation Promotes Smad7 Binding to gp130

**Figure 4:**
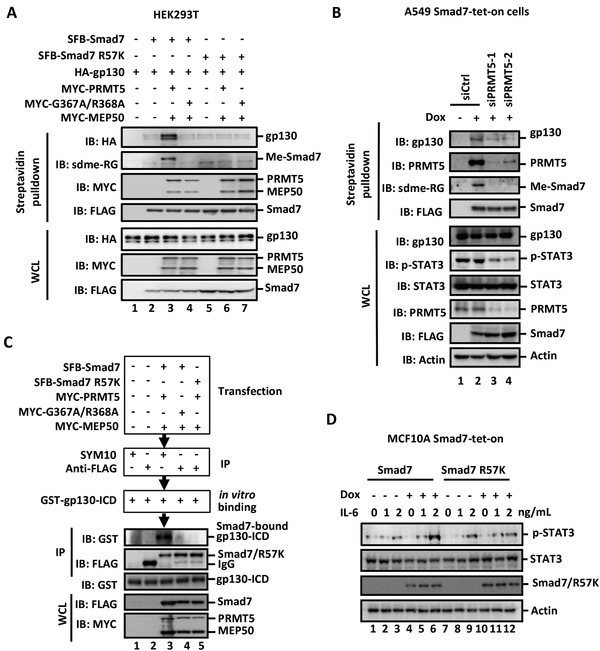
Arg methylation enhances Smad7 binding to gp130. A) Smad7 methylation increases its association with gp130. HEK293T cells were transfected with SFB‐Smad7 or Smad7 R57K mutant and HA‐gp130, together with MYC‐PRMT5/MEP50. Cell lysates were harvested and immunoprecipitated with Streptavidin beads. Western blotting analysis was done with appropriate antibodies. B) PRMT5 depletion blocks Smad7 methylation and its interaction with endogenous gp130. A549 tet‐on cells expressing SFB‐Smad7 were cultured with or without 1 µg mL^−1^ Dox for 3 d and then transfected with 40 × 10^−12^ m siPRMT5. Cell lysates were harvested and immunoprecipitated with streptavidin beads. Endogenous gp130 was detected from the immunoprecipitates by using Western blotting analysis. C) Methylated Smad7 binds more tightly to gp130. HEK293T cells were transfected with indicated expression plasmids for MYC‐PRMT5, MYC‐G367A/R368A, and MYC‐MEP50 as well as SFB‐Smad7 or SFB‐R57K. Dimethylated Smad7 was immunopurified using SYM10 antibody, while total Smad7 was retrieved using an‐FLAG antibody. Bacterially expressed GST‐gp130‐ICD was purified using glutathione‐sepharose and eluted with elution buffer (10 × 10^−3^ m glutathione, pH 8.0). In the in vitro binding experiments for evaluating the Smad7‐gp130 interaction, recombinant GST‐gp130‐ICD was added to the immunopurified Smad7. gp130‐ICD binding to immobilized Smad7 was analyzed by using Western blotting. D) Unmethylatable Smad7 R57K mutant loses its ability to potentiate STAT3 activation. MCF10A tet‐on cells stably expressing SFB‐Smad7 or Smad7 R57K were induced with 10 ng mL^−1^ Dox for 3 d, and treated with indicated concentrations of IL‐6. Cell lysates were collected and subject to Western blotting analysis.

As Smad7 potentiates STAT3 activation through direct binding to gp130, we speculated that methylation modification of Smad7 might affect this interaction. Indeed, methylated Smad7 exhibited a stronger binding ability to gp130 than unmethylated wildtype Smad7 or unmethylable R57K mutant (**Figure** **4**A). In addition, in Dox‐inducible A549 Smad7‐tet‐on cells (Figure S3G, Supporting Information), precipitated SFB‐Smad7 was clearly methylated as detected by adme‐RG antibody, and this methyl‐Smad7 could also pull down gp130 (Figure 4B). Remarkably, depletion of PRMT5 not only abolished methylation of Smad7, but also the Smad7‐gp130 association (Figure 4B).

To further investigate the role of R57 methylation in Smad7 binding to gp130, we immuno‐purified SFB‐Smad7 or SFB‐R57K that was coexpressed with MYC‐PRMT5 or MYC‐G367/R368A together with MYC‐MEP50 in HEK293T cells. As shown in Figure 4C, immunoprecipitation using SYM10 or FLAG antibodies could retrieve equal amounts of SFB‐Smad7 (lane 3–5). Notably, only methylated Smad7 interacted with gp130 (lane 3). In sharp contrast, un‐methylated Smad7 (i.e., in the presence of G367/R368A mutant) or unmethylatable Smad7 R57K mutant could not bind to gp130. In consistence, unlike wildtype Smad7, the R57K mutant lost its ability to augment IL‐6‐induced STAT3 activation (Figure 4D). These suggest that methylated Smad7 may possess a stronger binding ability to gp130.

### 2.5 PRMT5 Promotes STAT3 Signaling and Cellular Functions

**Figure 5:**
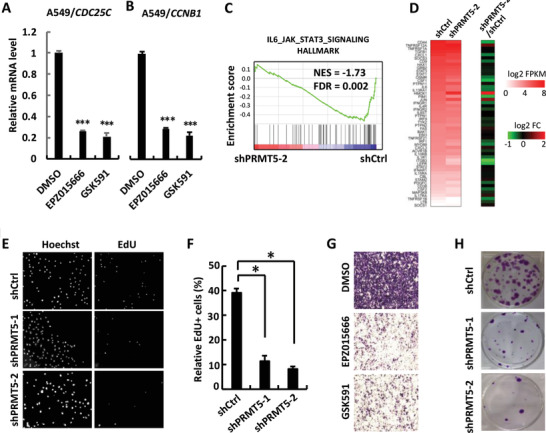
PRMT5 promotes STAT3 transcriptional and growth‐promoting responses. A) PRMT5 inhibition attenuates *CDC25C* expression in A549 cells. EPZ015666 or GSK591 (20 × 10^−6^ m) were added to A549 cells for 48 h. Cell lysates were harvested and analyzed by using qRT‐PCR to examine *CDC25C* mRNA levels. Data are shown as mean ± SD; *n* = 3. ***P < 0.001. B) PRMT5 inhibition attenuates *CCNB1* expression in A549 cells. Cell treatment, harvest, and qRT‐PCR analysis were done as described in Panel A. Data are shown as mean ± SD; *n* = 3. ***P < 0.001. C) PRMT5 deficiency disables IL‐6/STAT3 responsiveness. GSEA showed that downregulated genes in PRMT5‐depleted A549 cells (shPRMT5‐2) were highly enriched in the IL‐6/STAT3 signaling gene set. Red, upregulated genes; blue, downregulated genes. NES = ‐1.73, FDR *q* value = 0.002. D) Heatmap showing expression levels (log_2_FPKM; left) and relative expression changes (log_2_(shPRMT5‐2/shCtrl); right) of the IL‐6/STAT3 signaling genes. E) Depletion of PRMT5 reduces DNA synthesis. A549 cells stably expressing shPRMT5‐1 or shPRMT5‐2 or Control (shCtrl) were subject to EdU staining to determine DNA incorporating rate (RiboBio),20x. F) Statistic analysis of the result in panel E. Data are shown as mean ± SD; *n* = 3. 0.01 < *P < 0.05. G) Inhibition of PRMT5 attenuates invasiveness in A549 cells. A549 cells were treated with PRMT5 inhibitors EPZ015666 or GSK591 (20 × 10^−6^ m) for 2 d, starved overnight in FBS‐free medium. 1  ×  10^5^ cells were plated in a transwell chamber and stained with crystal violet after 12 h. Purple color indicates crystal violet staining of the invaded cell population. H) PRMT5 depletion blocks colony formation. A549 stable cells were subject to crystal violet staining and photography.

We next investigated whether PRMT5 regulates STAT3‐dependent transcriptional responses and growth‐promoting responses. Consistent with the ability of PRMT5 to promote STAT3 activation (Figure 1), qRT‐PCR analysis revealed that chemical inhibitors of PRMT5 profoundly decreased transcriptional activation of STAT3 target genes such as *CDC25C* (**Figure** **5**A)*, CCNB1* (Figure 5B), and *CDC25B* (Figure S4A, Supporting Information). In RNA‐seq experiments, gene set enrichment analysis (GSEA) revealed that *PRMT5* deficiency disabled IL‐6/STAT3 responsiveness as downregulated genes in PRMT5 knockdown A549 cells were significantly enriched in the IL‐6/STAT3 signaling gene set (shPRMT5‐1 vs shControl: NES Normalized Enrichment Score [NES] = ‐1.58, normalized *p* value [NOM *p*] = 0.016, false discovery rate *q* value [FDR *q*] = 0.011; shPRMT5‐2 vs shControl: NES = ‐1.73, NOM *p* < 0.001, FDR *q* = 0.002) (Figure 5C and Figure S4B, Supporting Information). Indeed, over half of the genes in the IL‐6/STAT3 signaling gene set were downregulated upon PRMT5 knockdown (Figure 5D). These results suggest that PRMT5 promotes STAT3 transcriptional responses.

To link PRMT5 to the well‐established growth‐promoting role of STAT3, we examined the functions of PRMT5 in cellular functions using cell‐based assays. STAT3 inhibitor stattic could significantly block STAT3 phosphorylation (Figure S4C, Supporting Information) and inhibit the growth of A549 cells (Figure S4D, Supporting Information). Knockdown of PRMT5 profoundly suppressed proliferative activities in A549 cells, as analyzed by using EdU staining (Figure 5E,F) or CCK‐8 assay (Figure S4E, Supporting Information) or colony formation assay (Figure 5H). In addition, PRMT5 inhibitors EPZ015666 and GSK591 strongly attenuated the ability of cell migration (Figure 5G). Meanwhile, overexpression of STAT3C, a constitutively activated STAT3 form,^[^ ^37^ ^]^ could largely reverse the inhibitory effect of PRMT5 deficiency on cell growth (Figure S4F, Supporting Information) and invasion (Figure S4G, Supporting Information). Taken together, these results demonstrate that PRMT5 promotes cell growth and migration through STAT3 signaling.

### 2.6 PRMT5 Promotes Lung Tumorigenesis

**Figure 6:**
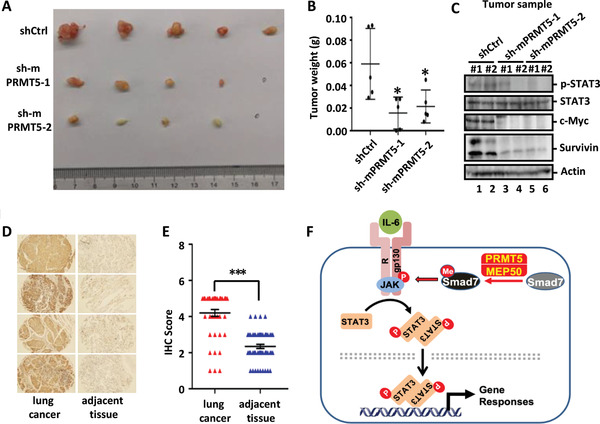
PRMT5 promotes lung tumorigenesis. A) Depletion of PRMT5 attenuates tumorigenesis. LLC cells stably expressing shControl or mouse sh‐mPRMT5‐1 or sh‐mPRMT5‐2 were subcutaneously injected into female nude mice. Ten days after implantation, tumors were dissected and photographed. B) Measurement of tumor weight in Panel A. Data are shown as mean ± SD; *n* = 5 for each group. 0.01 < *P < 0.05. C) PRMT5 depletion impairs STAT3 signaling in tumors. Tumor samples were analyzed by Western blotting to examine phosphorylated STAT3 (p‐STAT3) and STAT3 target gene products such as c‐Myc and Survivin. D) PRMT5 is highly expressed in nonsmall cell lung cancer tissues (NSCLC). NSCLC tissue microarray (Alenabio) was subject to immunohistochemistry (Servicebio) using PRMT5 antibody. E) Statistic analysis of IHC score in Panel D. Statistical analysis was performed using a two‐tailed Student's *t*‐test. Data are shown as mean ± SD. Lung cancer samples = 45. Normal lung tissue samples = 55. ***P < 0.001. F) A working model for PRMT5‐mediated STAT3 activation.

To further investigate the function of PRMT5 in tumor formation, a Lewis lung carcinoma (LLC) xenograft mouse model was used. In comparison to tumors derived from parental LLC cells, those with stable PRMT5 depletion using mouse‐specific shRNAs exhibited significantly reduced tumor growth and weight (**Figure** **6**A,B and Figure S5A, Supporting Information), demonstrating that knockdown of PRMT5 attenuated tumor development. Since PRMT5 depletion reduced expression of STAT3 target genes (Figure 5A–D and Figure S4A,B, Supporting Information), we analyzed the status of STAT3 tyrosine phosphorylation at Y705 (p‐STAT3) in mouse tumor samples. Notably, p‐STAT3 was reduced in shPRMT5 tumors in comparison to control tumors (Figure 6C). Accordingly, the expression of STAT3 target genes such as c‐Myc and survivin was nearly abolished in shPRMT5 tumors (Figure 6C). Collectively, our findings illustrate that PRMT5 contributes to human lung cancer cell proliferation and migration via modulating STAT3 signaling.

Immunohistochemical (IHC) analysis of tissue microarray of nonsmall cell lung cancer (NSCLC) showed that PRMT5 is markedly overexpressed in cancer cells compared to adjacent normal tissues (Figure 6D,E). Furthermore, elevated expression of both PRMT5 and cofactor MEP50 is strongly correlated with poorer prognosis in lung cancer patients from a public clinical database (http://www.kmplot.com) (Figure S5B,C, Supporting Information).

Taken all together, our results suggest that PRMT5‐mediated methylation of Smad7 enables its interaction with gp130, thus potentiating STAT3 activation, which consequently promotes cell proliferation and tumorigenesis (Figure 6F).

## 3 Discussion

PRMT5 is overexpressed in a large number of cancer types and promotes cell proliferation, migration, and survival.^[^ ^16^ ^]^ Besides controlling specific gene expression through methylation of histones, PRMT5 also targets certain transcription factors involving in cell signaling.^[^ ^16^ ^]^ Although PRMT5 was discovered as a JAK2‐binding protein, the role of PRMT5 in the JAK/STAT3 signaling pathway still remains unknown. Here we show that PRMT5 is critical for IL6‐induced STAT3 activation through the recently recognized Smad7‐gp130‐STAT3 axis.^[^ ^32^ ^]^ PRMT5 methylates Smad7, enhances the Smad7‐gp130 interaction, and promotes STAT3 signaling, whereas depletion or inhibition of PRMT5 consequently abolishes STAT3 activation and inhibits cell proliferation.

### 3.1 PRMT5 Activates IL‐6/STAT3 Signaling

Numerous evidence has clarified the importance of IL6/STAT3 signaling for cancer growth, metastasis, and maintenance of cancer stem cells.^[^ ^38^ ^]^ A variety of cytokines like IL‐6, epidermal growth factor (EGF), interferon (IFN) could induce STAT3 Tyr705 phosphorylation, a hallmark of JAK‐induced activation of STAT3. Some cells may have high levels of autocrine STAT3 inducers, while others can only trigger STAT3 activation in response to exogenous or paracrine cytokines/growth factors. We found that STAT3 is constitutively activated in human NSCLC A549 and H358 cells, which exhibit a high level of secreted IL‐6 (Figure S1B, Supporting Information). Addition of tocilizumab, a monoclonal antibody against IL‐6 receptor, to the growth medium of A549 cells could largely inhibit the endogenous STAT3 phosphorylation (Figure S1C, Supporting Information). In addition, mammary epithelial cell lines MCF10A and MCF7 exhibit STAT3 activation only when stimulated with IL‐6 in cell culture. Conditioned medium of A549 cells strongly stimulated STAT3 phosphorylation in MCF10A cells and this induction could be completely blocked by Tocilizumab (Figure S1D, Supporting Information). A variety of compounds against STAT3 signaling such as alantolactone,^[^ ^39^ ^]^ isocryptotanshinone,^[^ ^40^ ^]^ sinomenine^[^ ^41^ ^]^ suppress proliferation and invasion of A549 cells through disrupting STAT3 signaling pathway. Similar results were also observed for A549 cell proliferation in the presence of stattic (inhibitor specific to STAT3) (Figure S4D, Supporting Information). Thus, IL‐6/STAT3 signaling plays an important role in proliferation in A549 cells.

The critical evidence that connects PRMT5 to STAT3 activation comes from our observation that knockdown or inhibition of PRMT5 can significantly block STAT3 activation and cell responses to autocrine or exogenous IL‐6 (Figure 1 and Figure S1, Supporting Information). We found that A549 cells, which have a high level of autocrine IL‐6 and constitutive STAT3 activation, are sensitive to depletion or inhibition of PRMT5. Importantly, overexpression of STAT3C, a constitutively active mutant of STAT3,^[^ ^37^ ^]^ rescued the defect in cell proliferation and migration caused by PRMT5 inhibition (Figure S4F,G, Supporting Information). In addition, PRMT5 inhibition also blocks STAT3 activation induced by exogenous IL‐6 in MCF10A cells (Figure S1G, Supporting Information). These data support the notion that PRMT5 promotes cell growth and migration through maintaining or potentiating STAT3 activation.

### 3.2 Smad7 Connects PRMT5 to STAT3 Activation

How does PRMT5 activity eventually lead to STAT3 activation? It is very likely that PRMT5 must act at the level or upstream of STAT3 phosphorylation. Our study suggests an important role of Smad7 in mediates PRMT5‐induced STAT3 activation. Indeed, depletion of Smad7 strongly attenuates the positive effect of PRMT5 in IL‐6‐induced STAT3 phosphorylation (Figure 1H). Since the catalytic activity of PRMT5 is required for potentiating STAT3 phosphorylation, we speculate that protein methylation in addition to histone methylation may be involved in the regulation of STAT3 signaling. Indeed, inhibition of PRMT5 accompanies a reduction in the demethylation pattern that is recognized by an antibody against symmetric di‐methyl arginine motif (sdme‐RG) (Figure S1F,G, Supporting Information and Figure 1C). Because PRMT5 was first identified as a JAK2‐binding protein, we thus attempted to test possible methylation of JAK2 or any associated proteins in the gp130/JAK/STAT3 complexes. Although none of canonical components in the gp130/JAK/STAT3 pathway became methylated by PRMT5, Smad7 is clearly methylated by PRMT5 as methyl‐Smad7 protein can be recognized both in cells and in vitro (Figure 3A,E). We have previously reported a novel TGF‐*β*‐independent role of Smad7 enabling STAT3 activation.^[^ ^32^ ^]^ So the question is, does Smad7 methylation positively regulate its role in STAT3 activation?

We found that Smad7 exhibits a strong interaction with PRMT5 and MEP50 complex. MEP50, an essential partner of PRMT5, assists PRMT5 in recognizing its substrates. Smad7 could not be methylated by PRMT5 in the absence of MEP50 (Figure S3A, Supporting Information), which may explain why no methylation of Smad7 was previously reported by PRMT5.^[^ ^42^ ^]^ More detailed analysis demonstrates that PRMT5 interacts with the MH2 domain of Smad7 (Figure 2C and Figure S2I, Supporting Information) and dimethylates Arg‐57 in the MH1 region (Figure 3B). In sharp contrast, PRMT5 does not methylate Smad6, which has a divergent MH1 domain from Smad7 despite a highly conserved MH2 domain.^[^ ^35^ ^]^ Like Smad7, Smad7/6 chimera, but not the reverse Smad6/7 chimera, could also be methylated by PRMT5 (Figure S3D, Supporting Information). These observations suggest that PRMT5 methylates the MH1 of Smad7 although it binds to the MH2 domain. Interestingly, methylation of Smad7 at Arg57 possesses stronger binding to gp130 (Figure 4A), which may lead to better and stronger protection of gp130 from its endogenous inhibitors SHP2 and SOCS3.^[^ ^32^ ^]^

Smad7 is a well‐known negative feedback regulator in TGF‐*β* signaling. However, Smad7‐triggered STAT3 activation does not require functional TGF‐*β* receptors.^[^ ^32^ ^]^ Similarly, PRMT5 retains the ability to promote STAT3 activation in T*β*RI/ALK5‐null HaCaT cells (Figure S2H, Supporting Information). Although this further supports the non‐canonical function of Smad7, it does not exclude the possibility that Smad7 methylation also regulates TGF‐*β* signaling. A previous study has reported that Smad7 methylation by PRMT1 prevents its binding to T*β*RI/ALK5 and thus enables Smad3 activation, which is essential for TGF‐*β*‐induced epithelial‐mesenchymal transition.^[^ ^43^ ^]^ Since PRMT5 mediates symmetric di‐methyl modification on Smad7, which differs from PRMT1‐mediated mono‐methylation and asymmetric dimethylation, it is still worthy of future examination whether symmetric dimethylation also regulates Smad7 binding to T*β*RI/ALK5. If so, symmetric dimethylation serves as an important switch to facilitating TGF‐*β* signaling and gp130/STAT3 signaling.

Finally, our findings position PRMT5 as a critical regulator of gp130/STAT3 signaling to control the growth and development of human cancer, which provides an important mechanism for the oncogenic role of PRMT5. Given its enzymatic activity, PRMT5 is a druggable target. Indeed, PRMT5 inhibitors are in a number of clinical trials, for instance, to treat myelodysplastic syndrome (MDS), acute myeloid leukemia (AML), and solid tumors (including NSCLC) (https://clinicaltrials.gov/). It would be of great significance to investigate the effects of PRMT5 inhibitors in cancer types with aberrantly hyperactivated STAT3 signaling. Moreover, PRMT5‐mediated Smad7 methylation may also impact inflammation and cell pluripotency where gp130/STAT3 signaling is involved. It is conceivable that manipulating PRMT5 activity may also be used in therapies against inflammatory diseases and stem cell‐based regenerative medicine.

## 4 Experimental Section

##### Plasmids

The following mammalian expression plasmids have been previously described: FLAG‐Smad7,^[^ ^34^ ^]^ Smad7‐N, Smad7‐C,^[^ ^44^ ^]^ Smad6/7 and Smad7/6.^[^ ^33^ ^]^ SFB‐Smad7 generated by PCR was cloned into pRK5. MYC‐tagged and HA‐tagged PRMT5 and MEP50 were obtained by PCR and then cloned into pXF3HM (N‐terminal MYC tag) and pRK3HA (C‐terminal HA tag), respectively. All mammalian vectors are derived from pRK5 (Genentech). All mutants, including PRMT5 G367A/R368A, PRMT5r and Smad7 R57K were made by mutagenesis and confirmed by DNA sequencing. GST‐Smad7 was generated by PCR and cloned into pGEX4T1 (GST tag).

##### Antibodies and Reagents

Antibodies and their commercial sources are as follows: PRMT5 (ab109451) and gp130 (ab202850) from Abcam; p‐STAT3 (9145), STAT3 (9139), HA (3724) and sdme‐RG (13222) from Cell Signaling Technology; SYM10 (07‐412) from Merck millipore; FLAG (F3165), *β*‐actin (A5441), mouse IgG (I5381), and rabbit IgG(I5006) from Sigma‐Aldrich (USA); MYC (sc‐40) from Santa Cruz Biotechnology; Survivin (A19663) and c‐MYC (A1309) from Abclonal.

The following chemical compounds and recombinant proteins were commercially obtained: EPZ015666 (S7748) and GSK591 (S8111) from Selleck; Human IL‐6 (96‐200‐06‐20) from Peprotech and TGF‐*β* (TGFB1‐100) from StemRD.

##### Cell Culture and Transfection

HEK293T, LLC, and MCF7 cells were grown in DMEM (Corning) supplemented with 10% FBS (Gibco) at 37 °C in a humidified incubator with 5% CO_2_. A549 and H358 cells were cultured in PRMI 1640 (Corning) supplemented with 10% FBS. MCF10A were cultured in DMEM/F12 media (Corning) supplemented with 5% horse serum (Invitrogen), insulin (10 µg mL^−1^), EGF (20 ng mL^−1^), cholera toxin (100 ng mL^−1^) (Sigma‐Aldrich) and hydrocortisone (0.5 µg mL^−1^) (Sigma‐Aldrich). HEK293T cells were transfected with PEI (Polyscience, USA) and MCF7 cells with X‐treme GENE HP DNA (Roche, Switzerland). Stable cell lines were obtained by using lentiviral infection and selected in the presence puromycin/G418 at appropriate concentrations.

##### RNA Interference

siRNAs were commercially synthesized (Ribo‐Bio Co) and transfected into cells using Lipofectamine RNAiMAX (Invitrogen, USA). siRNA sequences targeting human genes were as below: siPRMT5‐1, GCCCAGTTTGAGATGCCTTAT; siPRMT5‐2, CCGCTATTGCACCTTGGAA; siSmad7, CCGTGCAGATCAGCTTTGT.

To generate stable knockdown of endogenous human PRMT5, mouse PRMT5 or Smad7, a lentiviral shRNA expression system was used. Two DNA fragments containing the target sequence of the corresponding target gene were cloned into the pLKO.1 lentivirus vector (Addgene #24150). Sequences are as below: human shPRMT5‐1, GCCATCACTCTTCCATGTTCT; human shPRMT5‐2, GCTATTGCACCTTGGAATTTC; mouse sh‐mPRMT5‐1, GGATGTGGTGGCATAACTTTC; mouse sh‐mPRMT5‐2, GCTAGAGAACTGGCAGTTTGA; human shSmad7, GTGCAGATCAGCTTTGTGA; nonspecific shCtrl, GGATAATGGTGATTGAGATGG. The pLKO.1‐shRNA plasmid together with the packaging plasmids was then transfected into HEK293T cells. After 48 h, viral supernatants were collected and filtered through a 0.45 µm filter, mixed with polybrene (10 µg mL^−1^) and added to the target cells for infection of 8 h. Cells were subject to puromycin (1 µg mL^−1^) selection for 3 d.

##### Quantitative RT‐PCR (qRT‐PCR)

Total RNAs (1 µg) were isolated from cells using TRIzol reagent (Sigma) and reverse‐transcribed to cDNA using transcriptor reverse transcriptase (Roche). cDNA was diluted and subject to qRT‐PCR analysis using SYBR green PCR Master Mix (Applied Biosystems). qRT‐PCR primers were listed as follows: h‐PRMT5‐Forward, 5’‐CTGTCTTCCATCCGCGTTTCA‐3’ and h‐PRMT5‐Reverse, 5’‐GCAGTAGGTCTGATCGTGTCTG‐3’; h‐Smad7‐Forward, 5’‐TTCCTCCGCTGAAACAGGG‐3’ and h‐Smad7‐Reverse, 5’‐CCTCCCAGTATGCCACCAC‐3’; h‐CDC25B‐Forward, 5’‐GCATGGAGAGTCTCATTAGTGC‐3’ and h‐CDC25B‐Reverse, 5’‐CTCCGCCTCCGCTTATTCT‐3’; h‐CDC25C‐Forward, 5’‐ATGACAATGGAAACTTGGTGGAC‐3’ and h‐CDC25C‐Reverse 5’‐GGAGCGATATAGGCCACTTCTG‐3’; h‐CyclinB1‐Forward, 5’‐AAGAGCTTTAAACTTTGGTCTGGG‐3’ and h‐CyclinB1‐Reverse, 5’‐CTTTGTAAGTCCTTGATTTACCATG‐3’.

##### Immunoprecipitation and Western Blotting

Co‐immunoprecipitation (co‐IP) was carried out using antibodies and protein A sepharose (GE Healthcare) or streptavidin beads. After several times washes, precipitated proteins were eluted in SDS loading buffer and separated by SDS‐PAGE, transferred onto PVDF membranes (Millipore), and detected in Western blotting with appropriate antibodies.

##### Mass Spectrometry

Protein gel bands were cut into small pieces and destained with destaining buffer (25 × 10^−3^ m NH_4_HCO_3_/25% methanol, pH 8.0). Gel pieces were reduced with 10 × 10^−3^ m DTT for 60 min at 56 °C and alkylation with 55 × 10^−3^ m iodoacetamide for 45 min. Gel pieces were washed with digestion buffer (50 × 10^−3^ m NH_4_HCO_3_, pH 8.0) twice, dehydrated with acetonitrile, and then dried in a speed‐vac. Gel pieces were rehydrated with trypsin solution (10 ng *μ*L^−1^ sequencing grade modified trypsin, 50 × 10^−3^ m NH_4_HCO_3_, pH 8.0) and incubated overnight at 37 °C. Digested peptides were extracted from gel pieces with elution buffer 1 (50% acetonitrile, 5% formic acid), elution buffer 2 (75% acetonitrile, 0.1% formic acid), sequentially. Gel pieces were dehydrated with acetonitrile twice, and all of the supernatants were combined. Peptides solution was dried in a speed vac, digested peptides were resuspended with 5% formic acid and desalted with StageTip.

Digested peptides were loaded on the analytical column (75 × 15cm, 1.9 µm C18, 1 µm tip) with Easy‐nLC 1200 system. Samples were analyzed with a 60 min gradient at a flow rate 300 nL min^−1^ as follows: 2–8% B for 2 min, 8–27% B for 43 min, 27–35% B for 8 min, 35–100% B for 3 min, 100% B for 4 min. Q Exactive HF‐X mass spectrometer was operated in data‐dependent mode with one full MS scan at *R* = 60 000 (m/z 200), followed by twenty HCD MS/MS scans at *R* = 15 000, NCE = 27, with an isolation width of 1.6 m/z. The AGC targets for MS1 and MS2 scans were 1 × 10^6^ and 5 × 10^4^, respectively, and the maximum injection time for MS1 and MS2 were 20 and 45 ms, respectively. Precursors of +1, +8 or above, or unassigned charge states were rejected; exclusion of isotopes was disabled; dynamic exclusion was set to 45 s. Mass spectrometry data were searched by MaxQuant.

##### GST Pulldown

GST pull‐down assays were carried out as described previously.^[^ ^45^ ^]^ In short, GST and GST‐fused Smad7 were prepared from *Escherichia coli* strain DE3 and purified using glutathione‐sepharose beads, then incubated with HEK293T cells expressing MYC‐tagged PRMT5/MEP50. PRMT5/MEP50 proteins associated with purified GST and GST‐fused Smad7 retrieved on the beads were analyzed by using Western blotting.

##### In Vitro Methylation Assay

HEK293T cells cultured in 10 cm dishes were transfected with SFB‐tagged Smad7 or MYC‐tagged PRMT5/MEP50 for 36 h. Cell lysates were immunoprecipitated by streptavidin beads or MYC antibody. The immunoprecipitated protein was incubated in the reaction buffer containing 20 × 10^−3^ m tris–HCl pH8, 0.01% Triton X‐100 (Sigma), 5 × 10^−3^ m DTT and 100 × 10^−6^ m SAM (Shenggong) at room temperature for 2 h. SDS loading buffer was added to end the reaction, and followed by Western blotting analysis.

##### Cell Counting Kit‐8 (CCK‐8) Assay

A549 cells were split into 96‐well plates (1 × 10^3^ cells, 100 µL per well, three replicates). 10 µL of the CCK‐8 solution was added to each well of the plate and continued to incubate in a humidified incubator for 2 h. Absorbance at 450 nm was measured using a microplate reader.

##### Colony Formation Assay

A549 cells were plated in a six‐well plate (1 × 10^3^ cells each well). Cell culture medium was refreshed every other day. After 2 weeks, cells were stained with 0.1% crystal violet and recorded.

##### Transwell Migration Assay

A 24‐well PET insert (BD Bioscience, USA) was used to test cell migration. A549 cells were serum‐starved overnight. 1 × 10^5^ cells in 200 µL FBS‐free medium were plated in transwell chambers, with 500 µL PRMI 1640 plus 10% FBS below the cell‐permeable membrane. After 12 h, cells inside the chamber were removed with a cotton swab, and migrated cells were gently washed with PBS for two times, fixed in 4% PFA for 20 min, and then stained with 0.1% crystal violet and photographed.

##### RNA‐Seq and Data Analysis

A549 stable cells were harvested for total RNA extraction with TRIzol (Invitrogen). RNA samples were then subject to mRNA‐Seq library preparation using the VAHTS mRNA‐seq V3 Library Prep Kit for Illumina (Vazyme). Barcoded libraries were pooled and sequenced on a HiSeq X Ten system (Illumina) to generate 150 bp paired‐end reads. Sequencing reads were mapped to the human genome (hg19) using Tophat v2.1.1. Only uniquely mapped reads (≈90% of total reads) were kept for subsequent analyses using Cufflinks v2.2.1, and gene expression levels were quantified as normalized FPKM (fragments per kilobase of exon per million mapped fragments). Genes with FPKM < 1 in all samples were excluded from the analyses. For the remaining genes, all FPKM values that are less than 1 were set to 1. Heatmaps were generated using the “pheatmap” package in R. GSEA was performed using the GSEA software (v3.0) with the hallmark gene sets (v7.0).^[^ ^29^ ^]^

##### In Vivo Tumor Formation Assay

All animal studies were approved by Zhejiang University Committee for Experimental Animal Studies and Ethics. A total of 2 × 10^6^ cells were suspended in a mixture of 100 µL phosphate‐buffered saline (PBS) and 100 µL Matrigel (BD Bioscience) and then injected subcutaneously into 6 week old female nude mice in the right flank. Ten days later, mice were sacrificed and tumors were excised and measured.

##### Statistical Analysis

Statistical analysis was performed using a two‐tailed Student's *t*‐test. Data are shown as mean ± SD.

## Conflict of Interest

The authors declare no conflict of interest.

## Supporting information

Supporting Information
