# Supplementary material for: PRMT5 Enables Robust STAT3 Activation via Arginine Symmetric Dimethylation of SMAD7
Source: Adv Sci (Weinh). 2021 Feb 24;8(10):2003047. doi: 10.1002/advs.202003047 (PMC8132155; doi:10.1002/advs.202003047)
Supplement: Supplementary file 1 — Supporting Information [file ADVS-8-2003047-s001.pdf]

© 2020 Wiley-VCH GmbH

ADVANCED SCIENCE
Open Access

Supporting Information

for *Adv. Sci.,* DOI: 10.1002/advs.202003047

PRMT5 Enables Robust STAT3 Activation via Arginine Symmetric Dimethylation of SMAD7

*Congcong Cai, Shuchen Gu, Yi Yu, Yezhang Zhu, HanChenxi Zhang, Bo Yuan, Li Shen, Bing Yang, and Xin-Hua Feng**

Supplementary Materials

Figure S1

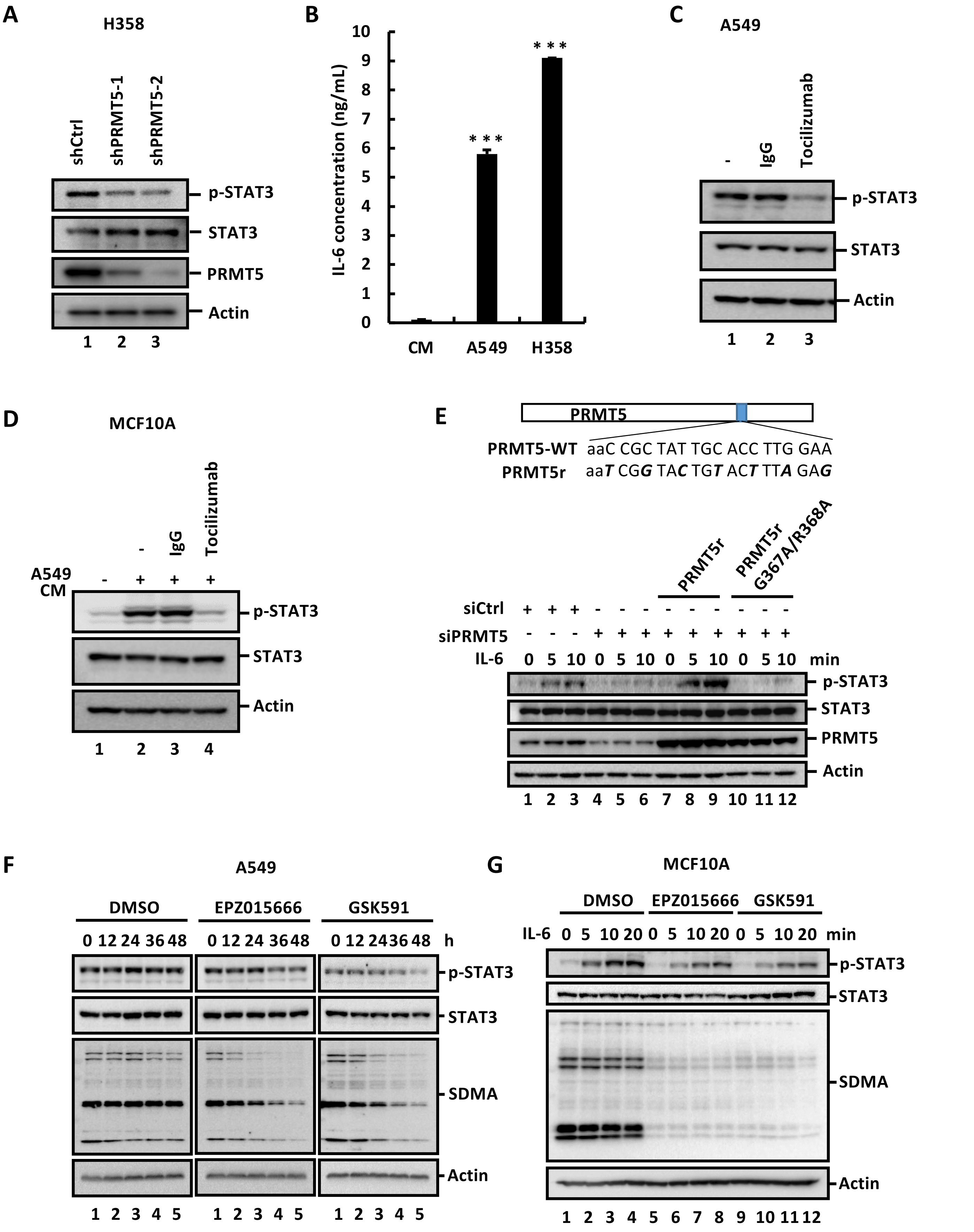
A
H358
shCtrl
shPRMT5-1
shPRMT5-2
p-STAT3
STAT3
PRMT5
Actin
1 2 3
B
IL-6 concentration (ng/mL)
0 1 2 3 4 5 6 7 8 9 10
***
***
CM
A549
H358
C
A549
-
IgG
Tocilizumab
p-STAT3
STAT3
Actin
1 2 3
D
MCF10A
-
IgG
Tocilizumab
A549 CM
- + + +
p-STAT3
STAT3
Actin
1 2 3 4
E
PRMT5
PRMT5-WT aaC CGC TAT TGC ACC TTG GAA
PRMT5r aaT CGG TAC TGT ACT TTA GAG
PRMT5r
PRMT5r G367A/R368A
siCtrl + + + - - - - - - - - -
siPRMT5 - - - + + + + + + + + +
IL-6 0 5 10 0 5 10 0 5 10 0 5 10 min
p-STAT3
STAT3
PRMT5
Actin
1 2 3 4 5 6 7 8 9 10 11 12
F
A549
DMSO
EPZ015666
GSK591
0 12 24 36 48
0 12 24 36 48
0 12 24 36 48 h
p-STAT3
STAT3
SDMA
Actin
1 2 3 4 5
1 2 3 4 5
1 2 3 4 5
G
MCF10A
DMSO
EPZ015666
GSK591
IL-6 0 5 10 20 0 5 10 20 0 5 10 20 min
p-STAT3
STAT3
SDMA
Actin
1 2 3 4 5 6 7 8 9 10 11 12

Figure S1. PRMT5 promotes IL-6-induced STAT3 activation dependent of its methyltransferase activity.

A. PRMT5 depletion dampens endogenous STAT3 activation in H358 cells. H358 cells stably expressing shPRMT5-1 or shPRMT5-2 or shControl (shCtrl) were harvested and analyzed by using Western blotting with indicated antibodies.

B. A549 and H358 cells secrete high levels of IL-6. Concentration of IL-6 in the conditioned medium of A549 or H358 cells ($1x10^6$ cells/mL) were measured using human IL-6 ELISA kit (R&D). Data are shown as mean ± SD; *n* = 3. ****P* < 0.001. CM means PRMI 1640 complete medium.

C. Autocrine IL-6 constitutively activates STAT3 in A549 cells. 80 μg/mL Anti-IL-6R antibody Tocilizumab was added to A549 cells for 24 h. Cell lysates were collected and subject to Western blotting analysis.

D. A549 cells-secreted IL-6 can be neutralized by Tocilizumab. A549-conditioned medium ($1x10^6$ cells) was collected and added to MCF10A cells pretreated with or without 5 μg/mL tocilizumab for 10 min. MCF10A cell lysates were harvested and subject to Western blotting analysis.

E. RNAi-resistant PRMT5r rescues STAT3 activation in PRMT5-depleted MCF10A cells. *Top,* schematic representation of the RNAi-resistant mutant of PRMT5 (PRMT5r) expression vector. siPRMT5 target sequence (encoding amino acids 533–539, above the nucleotide sequence) is indicated by capital letters. Mutations introduced are indicated by bold/italic letters. *Bottom*, MCF10A cells were transduced with lentiviral particles carrying RNAi-resistant PRMT5r or methyltransferase-dead G367A/R368A mutant. After 24 h, cells were transfected with 40 pm siPRMT5. 12 h later, cells were stimulated with IL-6 (10 ng/mL) for 5 or 10 min. Cell lysates were harvested and analyzed by using Western blotting. PRMT5r is the PRMT5 variant resistant to siPRMT5-2-mediated PRMT5 knockdown, and PRMT5r G367/R368A is the catalytic inactive variant resistant to siPRMT5-2.

F. PRMT5 inhibition dampens endogenous activation of STAT3 in A549 cells. A549 cells were treated with 20 μM of PRMT5 inhibitors EPZ015666 or GSK591 for the indicated time. Cell lysates were collected and subject to Western blotting analysis. SDMA indicates global arginine di-methylation.

G. PRMT5 inhibition attenuates IL-6-induced STAT3 activation in MCF10A cells. MCF10A cells were treated with EPZ015666 or GSK591 (20 μM) and stimulated with IL-6 (10 ng/ml) for indicated time. Cell lysates were analyzed by Western blotting with appropriate antibodies.

Figure S2

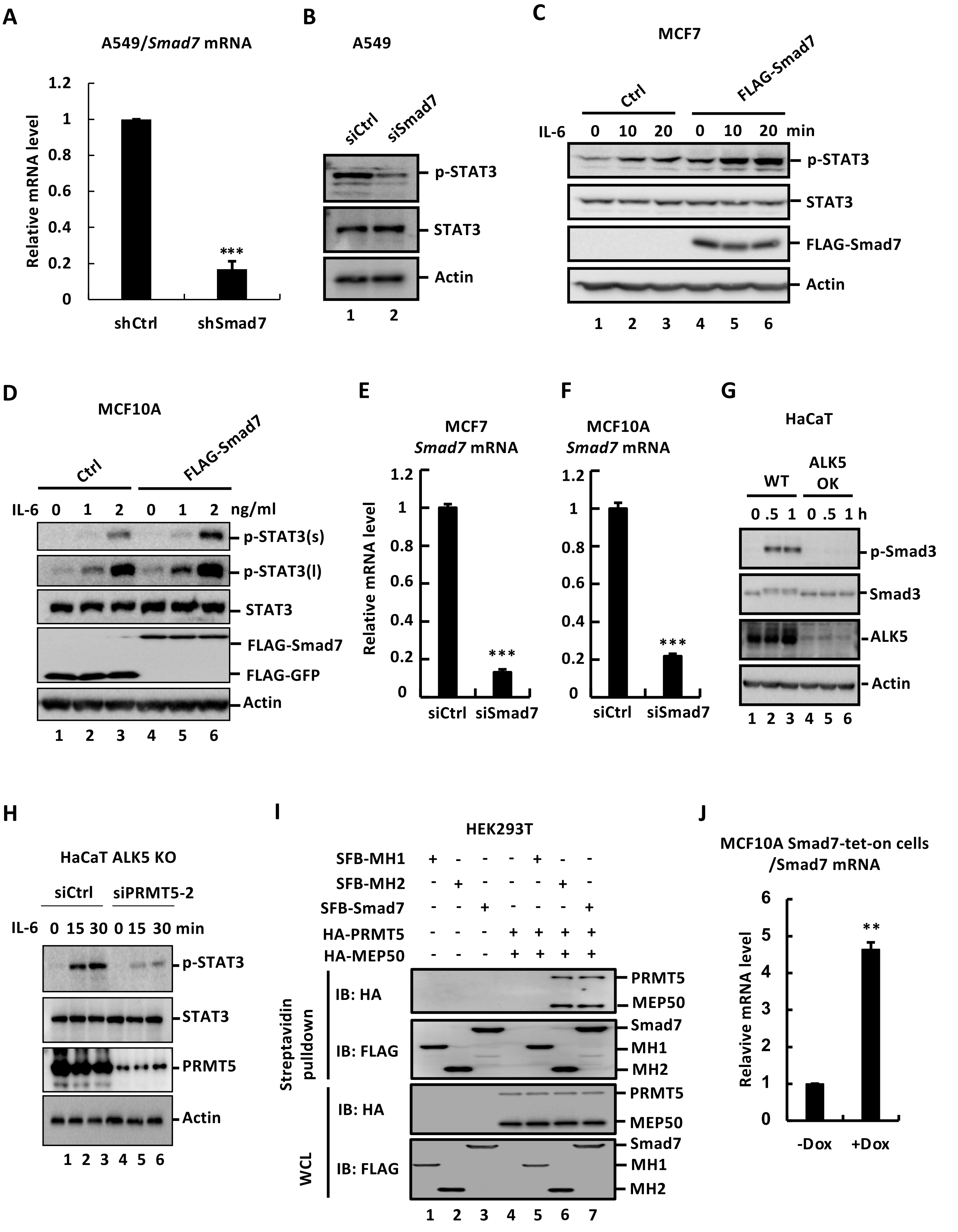
A
A549/Smad7 mRNA
Relative mRNA level
1.2
1
0.8
0.6
0.4
0.2
0
***
shCtrl
shSmad7
B
A549
siCtrl
siSmad7
p-STAT3
STAT3
Actin
1
2
C
MCF7
Ctrl
FLAG-Smad7
IL-6
0 10 20 0 10 20 min
p-STAT3
STAT3
FLAG-Smad7
Actin
1 2 3 4 5 6
D
MCF10A
Ctrl
FLAG-Smad7
IL-6
0 1 2 0 1 2 ng/ml
p-STAT3(s)
p-STAT3(l)
STAT3
FLAG-Smad7
FLAG-GFP
Actin
1 2 3 4 5 6
E
MCF7
Smad7 mRNA
Relative mRNA level
1.2
1
0.8
0.6
0.4
0.2
0
***
siCtrl
siSmad7
F
MCF10A
Smad7 mRNA
1.2
1
0.8
0.6
0.4
0.2
0
***
siCtrl
siSmad7
G
HaCaT
WT
ALK5 OK
0 .5 1 0 .5 1 h
p-Smad3
Smad3
ALK5
Actin
1 2 3 4 5 6
H
HaCaT ALK5 KO
siCtrl
siPRMT5-2
IL-6
0 15 30 0 15 30 min
p-STAT3
STAT3
PRMT5
Actin
1 2 3 4 5 6
I
HEK293T
SFB-MH1
+ - - - + - -
SFB-MH2
- + - - - + -
SFB-Smad7
- - + - - - +
HA-PRMT5
- - - + + + +
HA-MEP50
- - - + + + +
Streptavidin pulldown
IB: HA
PRMT5
MEP50
IB: FLAG
Smad7
MH1
MH2
WCL
IB: HA
PRMT5
MEP50
IB: FLAG
Smad7
MH1
MH2
1 2 3 4 5 6 7
J
MCF10A Smad7-tet-on cells
/Smad7 mRNA
Relavive mRNA level
6
5
4
3
2
1
0
**
-Dox
+Dox

Figure S2. Smad7 drives IL-6-induced STAT3 activation.

A. Smad7 is efficiently knocked down by shRNA against Smad7. RNA was extracted from A549 stably expressing shControl (shCtrl) and sh-Smad7 and subject to qRT-PCR to examine mRNA levels of *Smad7*. Data are shown as mean ± SD; $n = 3$. ***$P < 0.001$.

B. Depletion of Smad7 blocks STAT3 activation in A549 cells. 40 pm siRNA specifically targeting *Smad7* were transfected into A549 cells. After 48 h, cell lysates were subject to Western blotting analysis using indicated antibodies.

C. Smad7 potentiates IL-6-induced STAT3 activation in MCF7 cells. MCF7 cells were transfected with FLAG-tagged Smad7. After 36 h, cells were stimulated with IL-6 (50 ng/ml) for indicated time. Cell lysates were subject to Western blotting analysis.

D. Smad7 potentiates IL-6-induced STAT3 activation in MCF10A cells. MCF10A cells stably expressing FLAG-GFP or FLAG-Smad7 were treated with indicated concentrations of IL-6. Fifteen min later, cell lysates were harvested and subject to Western blotting analysis.

E. Smad7 is efficiently knocked down by siRNA against Smad7 in MCF7 cells. MCF7 were transiently transfected with 40 pm siRNA specific to Smad7. After 48 h, cells were harvested to extract RNAs. qRT-PCR was carried out to examine mRNA levels of Smad7. Data are shown as mean ± SD; $n = 3$. ***$P < 0.001$.

F. Smad7 is efficiently knocked down by siRNA against Smad7 in MCF10A cells. Cell transfection, RNA extraction and qRT-PCR were done as described in Panel E. Data are shown as mean ± SD; $n = 3$. ***$P < 0.001$.

G. ALK5-null HaCaT cells are not responsive to TGF-β. HaCaT ALK5-KO cells were treated with TGF-β (2 ng/ml) for indicated time. Cell lysates were analyzed by Western blotting with appropriate antibodies.

H. PRMT5 promotes STAT3 activation independent of TGF-β receptor. HaCaT ALK5-KO cells were transfected with 40 pm siRNA specific to PRMT5 and treated with IL-6 (50 ng/ml) for indicated time. Cell lysates were analyzed by Western blotting with appropriate antibodies.

I. PRMT5/MEP50 interact with Smad7 in the MH2 domain. HEK293T cell transfection and Western blotting analysis were done as described in Figure 2C.

J. Inducible stable expression of Smad7. MCF10A Smad7-tet-on cells, which stably express SFB-Smad7 in response to Dox, were cultured with or without Dox (500 ng/ml) for 72 h. Cell lysates were subject to RNA extraction and subsequent qRT-PCR. Data are shown as mean ± SD; n = 3. $0.001 <$ **$P < 0.01$.

Figure S3

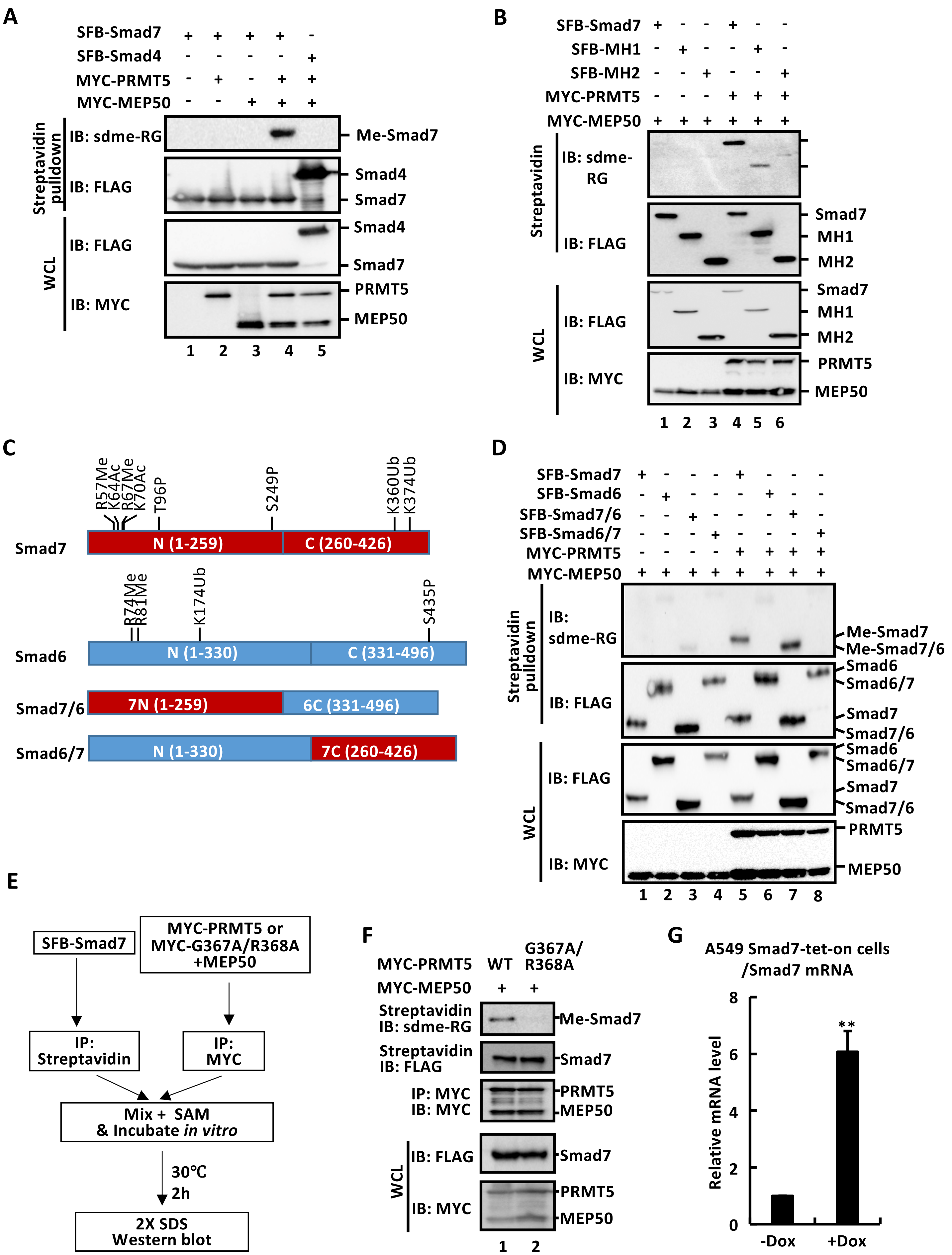

A
SFB-Smad7
SFB-Smad4
MYC-PRMT5
MYC-MEP50
Streptavidin pulldown
IB: sdme-RG
IB: FLAG
WCL
IB: FLAG
IB: MYC
Me-Smad7
Smad4
Smad7
PRMT5
MEP50
1 2 3 4 5
B
SFB-Smad7
SFB-MH1
SFB-MH2
MYC-PRMT5
MYC-MEP50
Streptavidin
IB: sdme-RG
IB: FLAG
WCL
IB: FLAG
IB: MYC
Smad7
MH1
MH2
PRMT5
MEP50
1 2 3 4 5 6
C
R57Me
K64Ac
R67Me
K70Ac
T96P
S249P
K360Ub
K374Ub
Smad7
N (1-259)
C (260-426)
R74Me
R81Me
K174Ub
S435P
Smad6
N (1-330)
C (331-496)
Smad7/6
7N (1-259)
6C (331-496)
Smad6/7
N (1-330)
7C (260-426)
D
SFB-Smad7
SFB-Smad6
SFB-Smad7/6
SFB-Smad6/7
MYC-PRMT5
MYC-MEP50
Streptavidin pulldown
IB: sdme-RG
IB: FLAG
WCL
IB: FLAG
IB: MYC
Me-Smad7
Me-Smad7/6
Smad6
Smad6/7
Smad7
Smad7/6
PRMT5
MEP50
1 2 3 4 5 6 7 8
E
SFB-Smad7
MYC-PRMT5 or MYC-G367A/R368A +MEP50
IP: Streptavidin
IP: MYC
Mix + SAM & Incubate in vitro
30℃ 2h
2X SDS Western blot
F
MYC-PRMT5
WT
G367A/ R368A
MYC-MEP50
Streptavidin IB: sdme-RG
Streptavidin IB: FLAG
IP: MYC IB: MYC
WCL
IB: FLAG
IB: MYC
Me-Smad7
Smad7
PRMT5
MEP50
1 2
G
A549 Smad7-tet-on cells /Smad7 mRNA
Relative mRNA level
0
2
4
6
8
**
-Dox
+Dox

Figure S3. PRMT5 methylates Smad7 in the MH1 domain.

A. PRMT5 depends on MEP50 to methylate Smad7. HEK293T cells were transfected with indicated expression plasmids. Experiments were performed as described in Fig. 3A.

B. PRMT5 methylates Smad7 in the N-terminal MH1 domain. HEK293T cells were transfected with MYC-tagged PRMT5/MEP50 and SFB-tagged Smad7 wildtype or mutant. Cell lysates were harvested and precipitated with Streptavidin beads. Arginine di-methylated Smad7 was detected by using Western blotting analysis.

C. Schematic presentation of Smad6, Smad7 and chimera. Domain structures and known modifications are shown.

D. PRMT5 methylates Smad7 and Smad7/Smad6 chimera. HEK293T cell transfection, Streptavidin precipitatation and Western blotting analysis were done as described in Panel A.

E. Flow chart of the *in vitro* methylation assay.

F. PRMT5 methylates Smad7 *in vitro*. SFB-Smad7 was expressed in HEK293T cells and purified using Streptavidin beads. MYC-PRMT5 or MYC-G367A/R368A mutant were co-expressd with MEP50 in HEK293T cells and purified using MYC antibody, which presumably retrieve the PRMT5/MEP50 complex. SFB-Smad7 and MYC-PRMT5/MEP50 or mutant were then incubated in the presence of S- adenosyl-methionine to allow Arginine di-methylation (Me). Methyl Smad7 was detected by Western blotting analysis.

G. Dox-induced SFB-Smad7 expression in A549 cells. A549 Smad7-tet-on cell culture, treatment, cell lysis and qRT-PCR were done as in Fig S2I. Dox concentration was 1000 ng/mL.

Figure S4

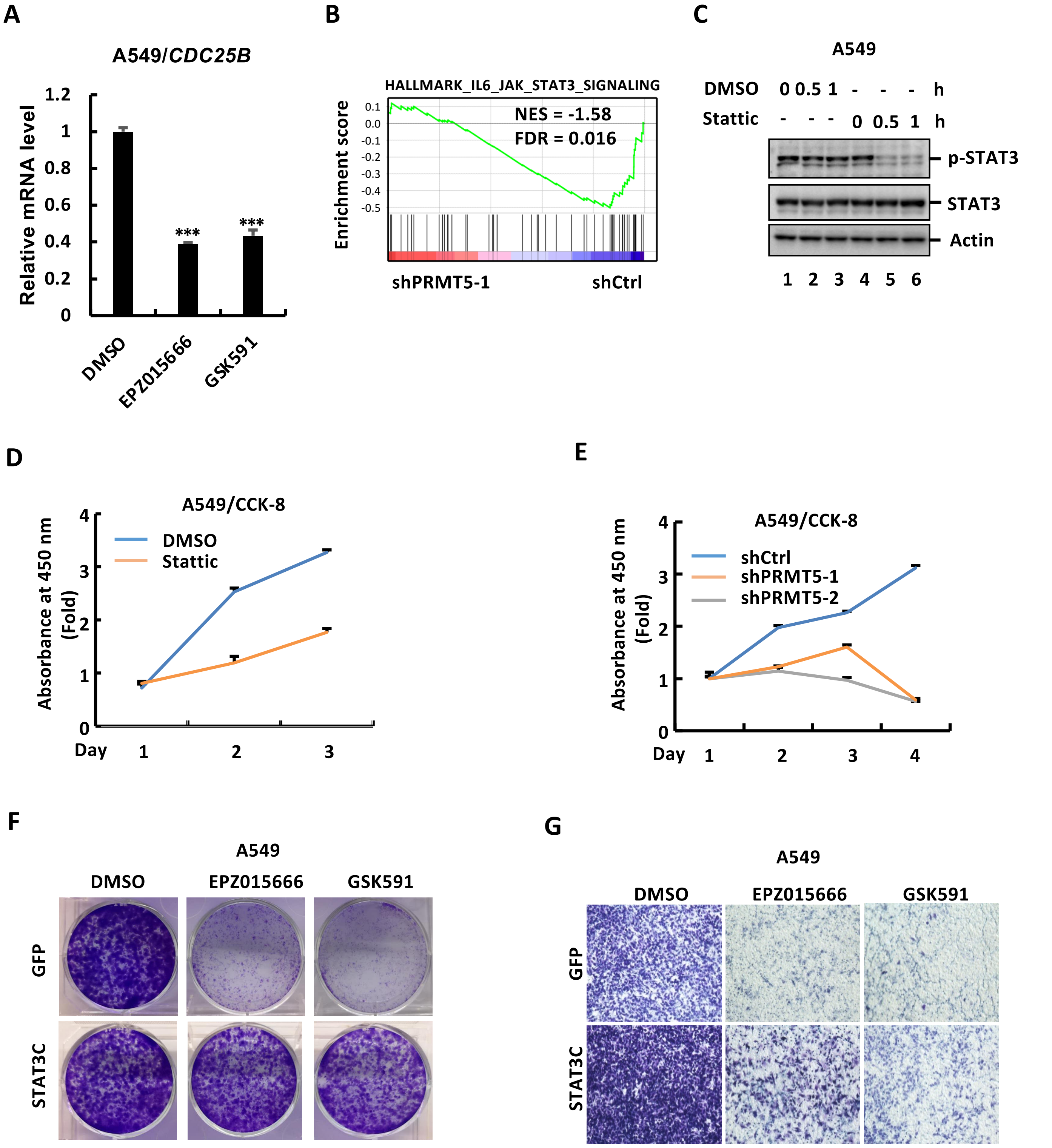
A
A549/CDC25B
Relative mRNA level
1.2
1
0.8
0.6
0.4
0.2
0
***
***
DMSO
EPZ015666
GSK591
B
HALLMARK_IL6_JAK_STAT3_SIGNALING
Enrichment score
NES = -1.58
FDR = 0.016
shPRMT5-1
shCtrl
C
A549
DMSO 0 0.5 1 - - - h
Stattic - - - 0 0.5 1 h
p-STAT3
STAT3
Actin
1 2 3 4 5 6
D
A549/CCK-8
Absorbance at 450 nm (Fold)
DMSO
Stattic
Day 1 2 3
E
A549/CCK-8
Absorbance at 450 nm (Fold)
shCtrl
shPRMT5-1
shPRMT5-2
Day 1 2 3 4
F
A549
DMSO
EPZ015666
GSK591
GFP
STAT3C
G
A549
DMSO
EPZ015666
GSK591
GFP
STAT3C

Figure S4. STAT3 largely mediates the effect of PRMT5 in A549 cells.

A. PRMT5 inhibition attenuates *CDC25B* expression in A549 cells. Two specific PRMT5 inhibitors EPZ015666 or GSK591 (20 μM) were added to A549 cells for 48 h. Cell lysates were subject to qRT-PCR to examine mRNA levels of *CDC25B*. Data are shown as mean ± SD; $n = 3$. ***$P < 0.001$.

B. PRMT5 deficiency disabled IL-6/STAT3 responsiveness. GSEA was done as described in Fig 5C. Red, upregulated genes; blue, downregulated genes. NES = -1.58, FDR *q* value = 0.016.

C. Stattic inhibits STAT3 activation. DMSO or 10 μM Stattic was added to A549 cells for indicated times. Cell lysates were subject to Western blotting analysis.

D. A549 cells depend on STAT3 activation for proliferation. A549 cells were treated with 10 μM Stattic and subject to cell growth assay using CCK-8 kit. Data are shown as mean ± SD.

E. PRMT5 depletion attenuates cell growth. A549 cells stably expressing shControl (shCtrl), shPRMT5-1 or shPRMT5-2 in a 96 well plate ($1x10^3$ cells/well) were subject to cell growth assay using CCK-8 kit with absorbance at 450 nm being recorded. Data are shown as mean ± SD.

F. STAT3C rescued the inhibitory effect of PRMT5 inhibitors on cell growth. A549 cells stably expressing GFP or STAT3C in 6-well plates ($1x10^3$ cells/well) were treated with PRMT5 inhibitors EPZ015666 or GSK591 (20 μM) for 14 d with 2 d interval. Cells were subject to crystal violet staining and photography.

G. STAT3C rescued the inhibitory effect of PRMT5 inhibitors on cell migration. A549 cells stably expressing GFP or STAT3C were treated with PRMT5 inhibitors EPZ015666 or GSK591 (20 μM) for 2 d. Experiments were performed as described in Fig 5G.

Figure S5

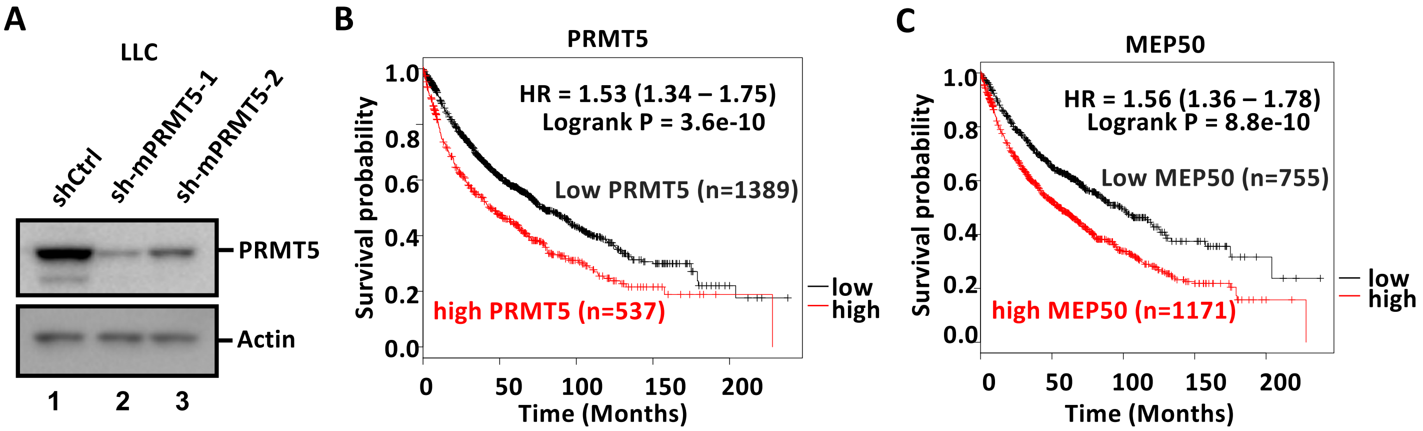

Figure S5. PRMT5 and MEP50 are correlated with poor prognosis.

A. shRNA-mediated knockdown of PRMT5 in mouse Lewis lung carcinoma. Mouse cells stably expressing shControl (shCtrl), sh-mPRMT5-1 or sh-mPRMT5-2 were collected and subject to Western blotting analysis.

B. High PRMT5 expression is correlated with poor prognosis. Kaplan-Meier survival probability plot for high (red) versus low (black) expression of PRMT5 in lung cancer was done using data from http://www.kmplot.com.

C. High MEP50 expression is correlated with poor prognosis. Data analysis was similarly done as in Panel B.
